# Production of Bacterial Cellulose by *Gluconacetobacter hansenii* Using Corn Steep Liquor As Nutrient Sources

**DOI:** 10.3389/fmicb.2017.02027

**Published:** 2017-10-17

**Authors:** Andrea F. S. Costa, Fabíola C. G. Almeida, Glória M. Vinhas, Leonie A. Sarubbo

**Affiliations:** ^1^Northeast Biotechnology Network, Federal Rural University of Pernambuco, Recife, Brazil; ^2^Design and Communication Center, Academic Region Agreste Center, Federal University of Pernambuco, Caruaru, Brazil; ^3^Center of Sciences and Technology, Catholic University of Pernambuco, Recife, Brazil; ^4^Advanced Institute of Technology and Innovation, Recife, Brazil; ^5^Department of Chemical Engineering, Technology and Geosciences Center, Federal University of Pernambuco, Recife, Brazil

**Keywords:** bacterial cellulose, *Gluconacetobacter hansenii*, carbon source, nitrogen source, waste

## Abstract

Cellulose is mainly produced by plants, although many bacteria, especially those belonging to the genus *Gluconacetobacter*, produce a very peculiar form of cellulose with mechanical and structural properties that can be exploited in numerous applications. However, the production cost of bacterial cellulose (BC) is very high to the use of expensive culture media, poor yields, downstream processing, and operating costs. Thus, the purpose of this work was to evaluate the use of industrial residues as nutrients for the production of BC by *Gluconacetobacter hansenii* UCP1619. BC pellicles were synthesized using the Hestrin–Schramm (HS) medium and alternative media formulated with different carbon (sugarcane molasses and acetylated glucose) and nitrogen sources [yeast extract, peptone, and corn steep liquor (CSL)]. A jeans laundry was also tested. None of the tested sources (beside CSL) worked as carbon and nutrient substitute. The alternative medium formulated with 1.5% glucose and 2.5% CSL led to the highest yield in terms of dry and hydrated mass. The BC mass produced in the alternative culture medium corresponded to 73% of that achieved with the HS culture medium. The BC pellicles demonstrated a high concentration of microfibrils and nanofibrils forming a homogenous, compact, and three-dimensional structure. The biopolymer produced in the alternative medium had greater thermal stability, as degradation began at 240°C, while degradation of the biopolymer produced in the HS medium began at 195°C. Both biopolymers exhibited high crystallinity. The mechanical tensile test revealed the maximum breaking strength and the elongation of the break of hydrated and dry pellicles. The dry BC film supported up to 48 MPa of the breaking strength and exhibited greater than 96.98% stiffness in comparison with the hydrated film. The dry film supported up to 48 MPa of the breaking strength and exhibited greater than 96.98% stiffness in comparison with the hydrated film. The values obtained for the Young’s modulus in the mechanical tests in the hydrated samples indicated low values for the variable rigidity. The presence of water in the interior and between the nanofibers of the hydrated BC only favored the results for the elasticity, which was 56.37% higher when compared to the dry biomaterial.

## Introduction

Cellulose is a highly abundant natural polymer produced mainly by plants (Gomes et al., 2013). Growing demand for products containing vegetal cellulose has increased the consumption of wood, thereby contributing to deforestation, which is a global environmental issue (Park et al., 2014). However, several bacteria also produce cellulose. Brown (1886) identified the growth of an unbranched pellicle produced by bacteria with a structure that was chemically equivalent to vegetal cellulose (Hestrin and Schramm, 1954; Klemm et al., 2001; Donini et al., 2010; Rangaswamy et al., 2015).

Bacterial cellulose (BC) is produced by bacteria from the genera *Gluconacetobacter, Sarcina*, and *Agrobacterium* (Donini et al., 2010; Huang et al., 2014) in both synthetic and non-synthetic media through oxidative fermentation (Esa et al., 2014). The aerobic gram-negative bacterium *G. hansenii* is one the most studied BC producer that assimilate various sugars and yields high levels of cellulose in liquid medium (Ross et al., 1991; Sani and Dahman, 2010; Moosavi-Nasab and Yousefi, 2011). During the synthetic process, the glucose chains produced inside the bacterial body extrude out through tiny pores present on their cell envelope. The glucose chains then form microfibrils that further aggregate to form cellulose ribbons. These ribbons generate a web-shaped network structure with plenty of empty spaces between the fibers. The well-separated nanofibrils of BC create an expanded surface area and highly porous matrix (Shah et al., 2013; Esa et al., 2014). The basic fibril structure consists of a β-1→4 glucan chain with the following molecular formula: (C_6_H_10_O_5_)n. The chains are held together by hydrogen bonds (Ul-Islam et al., 2012). BC microfibrils are approximately 100-fold smaller than the fibrils of vegetal cellulose (Chawla et al., 2009; Gayathry and Gopalaswamy, 2014). The fibrous network of BC consists of well-arranged, three-dimensional nanofibers resulting in the formation of hydrogel film with a large surface area and considerable porosity (Esa et al., 2014). As it is not associated with lignin or hemicelluloses as in vegetal cellulose, BC cellulose is purer; moreover, the three-dimensional nanofibril network has a high-water absorption capacity and tensile strength (Wu et al., 2014).

Different forms of BC are produced when the fermentation process is conducted under static, agitated, or stirred conditions. A three-dimensional interconnected reticular pellicle is produced under static fermentation, whereas agitated and stirred conditions produce an irregularly shaped, sphere-like cellulose particle (Tanskul et al., 2013). Cellulose formation under static conditions is regulated by the air supply from the surface of the medium, and the yield depends on the concentration of the carbon source (Budhiono et al., 1999). A longer growth time leads to an increase in the formation of BC through hydrogen and C–H bonding (Sheykhnazaria et al., 2011). However, BC production reaches a limit when the pellicle grows downward and entraps the bacteria, which then become inactive due to insufficient oxygen (Borzani and Souza, 1995). On the industrial scale, a semi-continuous rather than continuous process is recommended under static conditions to increase BC production (Çakar et al., 2014).

Bacterial cellulose (BC) forms as a white leathery pellicle at the air–liquid interface. Although its molecular structure is identical to that of vegetal cellulose, BC has higher degrees of purity, polymerization, crystallinity, tensile strength, water absorption, water retaining capacity, and biological adaptability, offering biocompatibility, biodegradability, and renewability. These unique properties result in a wide range of applications. In the field of biomedicine, BC can be used in wound dressings for the recuperation of burned skin, as a membrane for the delivery of dermal drugs, as an artificial blood vessel in microsurgical procedures, and as a polymeric scaffold for the repair of cartilage and bone (Klemm et al., 2001; Svensson et al., 2005; Czaja et al., 2006, 2007; Puppi et al., 2010; Trovatti et al., 2011). BC also has applications in other technological fields as membranes for audio devices, in the production of electronic paper, as reinforcement material in transparent/translucent nanocomposites and for coating paper, as well as other uses (Iguchi et al., 2000; Shah and Brown, 2005; Nogi and Yano, 2008; Fernandes et al., 2009; Pascoal Neto et al., 2011; Gomes et al., 2013).

However, the high cost of fermentation media has limited the industrial production of BC, as such media account for 30% of the total production cost (Rivas et al., 2004). Thus, finding new cost-effective culture media to achieve the highest yield of BC in large-scale industrial applications is paramount and requires new carbon and nitrogen sources. The Hestrin–Schramm (HS) medium is commonly used in the cultivation of BC. However, this medium is expensive and requires additional products, as glucose, yeast, peptone, etc. Thus, wider applications of BC depend on practical considerations with regard to scale-up capability and production costs (Kongruang, 2008; Cavka et al., 2013; Huang et al., 2014). In recent years, studies have focused on a variety of cellulose-producing bacterial strains, inexpensive nutrient sources, and supplementary materials for the production of inexpensive BC (Kiziltas et al., 2015).

Different waste products from agricultural and industrial activities have been investigated as a means to improve the yield and decrease the cost of BC production, such as dry olive mill residue (Gomes et al., 2013), sugarcane molasses (Çakar et al., 2014; Tyagi and Suresh, 2016), waste beer yeast (Lin et al., 2014), wastewater from candy processing (Li et al., 2015), wood sugars (Kiziltas et al., 2015), waste from fruit processing (Hungund and Gupta, 2010), lipid fermentation wastewater (Huang et al., 2014), rice bark (Goelzer et al., 2009), konjac powder (Hong and Qiu, 2008), cotton-based textile waste (Jeihanipour and Taherzadeh, 2009), and coffee bean husks (Rani and Appaiah, 2013). The use of such materials could improve the sustainability of BC production as well as reduce environmental pollution associated with the disposal of industrial waste. The potential of BC goes beyond existing applications, especially if produced in large amounts from inexpensive feedstock. Such applications may include specialty textiles, packaging, and advanced functional materials (Cavka et al., 2013).

The aim of the present study was to investigate the effects of different carbon and nitrogen sources to formulate a general, simple, inexpensive medium for the production of BC when compared to the HS standard medium. Analyses were performed of the yield, morphology, and structure of the BC hydrated and dry pellicles produced. The nanofibers produced in the selected medium were then characterized using X-ray diffraction (XRD), scanning electron microscopy (SEM), thermogravimetric analysis (TGA), and mechanical testing.

## Materials and Methods

### Materials

Glucose, peptone, yeast extract, citric acid monohydrate, agar, and sodium hydroxide were purchased from Merck Ltd., United States. Corn steep liquor (CSL), sugarcane molasses, and laundry effluent of jeans processing were procured from local companies in the state of Pernambuco, Brazil. The jeans laundry effluent was characterized by macroscopic (visual), physical–chemical (pH, conductivity, turbidity, and color by spectrophotometry at 455 nm), and microbiological (bacteria, filamentous fungi, and yeasts counts) analyses and presented favorable results to allow the possible obtaining of a colored BC pellicle.

The effluent sample had the following characteristics: pH in the range of 5.9–7.0, electrical conductivity of 208 μS/cm, COD of 608 mg O_2_/L, and turbidity of 19.5 UNT. The effluent presented high values for the color parameter. The apparent color ranged from 1240 to 1360 mg Pt/L and true color from 229 to 235 mg Pt/L. The following maximum CFU/mL counts were obtained as follows: 3 × 10^7^ for bacteria, 2 × 10^2^ for yeasts, and 4 × 10 for filamentous fungi. The effluent was sterilized by autoclaving at 121°C for 20 min prior to use.

### Microorganism

For BC production, a strain of *Gluconacetobacter hansenii* UCP1619, obtained from the culture collection of Nucleus of Resource in Environmental Sciences, Catholic University of Pernambuco, Brazil, was used. The standard culture medium used in the experiments was the HS described by Hestrin and Schramm (1954) and modified by Hungund and Gupta (2010). The liquid medium contained 2.0% glucose (w/v), 0.5% yeast extract (w/v), 0.5% peptone, 0.27% Na_2_HPO_4_ (w/v), and 0.15% citric acid (v/v). To formulate desolid medium, 1.8% agar (w/v) was added. The pH was adjusted to 5.0 using NaOH 1.0 M. The medium was stored at 4°C in a refrigerator and sub-cultured every 2 months for inoculum development.

### Culture Conditions

The inoculum culture was prepared by transferring the cellulose film containing the *G. hansenii* adhered cells stored at -80°C in HS agar to the liquid HS medium, followed by static cultivation at 30°C for 2 days. The statically grown culture was then shaken vigorously to homogenize the cell distribution in the inoculum. The resulting cell suspension (3 mL) was inoculated in a semi-capped glass vessel (250 mL) containing 100 mL of the different liquid media, as described below, and then statically incubated at 30°C for 10 days in triplicate experiments (Gomes et al., 2013; Wu et al., 2014). After cultivation, the BC pellicles were sent for cleaning, purification as well as the determination of thickness, hydrated mass, and dry mass, as it will be described in the Section “Harvesting and Weighing of BC.” The experiments were carried out under sterile conditions.

### Effect of Carbon and Nitrogen Sources on BC Production

Different carbon (glucose, acetylated glucose, and molasses) and nitrogen (yeast extract, peptone, and CSL) sources were used in the HS medium for the comparison of the total yield (dry mass) of cellulose produced by the bacterial isolate. A jeans laundry effluent was also tested. **Table 1** lists the compositions of the different media (pH adjusted to 5–6) used for cellulose production.

**Table 1:** Composition of media used for production of BC.

Media components	Culture medium									
	HS	Modified HS media (alternative media)								
		1	2	3	4	5	6	7	8	9
Glucose (g/L)	20	–	–	20	–	20	–	20	–	–
Peptone (g/L)	5.0	5.0	5.0	–	–	5.0	5.0	–	–	5.0
Yeast extract (g/L)	5.0	5.0	5.0	–	–	5.0	5.0	–	–	5.0
Disodium phosphate (g/L)	2.7	2.7	2.7	2.7	2.7	2.7	2.7	2.7	2.7	2.7
Citric acid monohydrate (g/L)	1.5	1.5	1.5	1.5	1.5	1.5	1.5	1.5	1.5	1.5
Sugarcane molasses (g/L)	–	–	20	–	20	–	20	–	20	–
Corn steep liquor (CSL) (g/L)	–	–	–	10	10	–	–	10	10	–
Acetylated glucose (g/L)	–	20	–	–	–	–	–	–	–	20
Distilled water (L)	1.0	1.0	1.0	1.0	1.0	–	–	–	–	–
Jeans laundry effluent (L)	–	–	–	–	–	1.0	1.0	1.0	1.0	1.0

### Maximization of BC Production in Selected Alternative Medium

After selection of the alternative medium (modified HS medium) consisting of 20 g/L of glucose and 10 g/L of CSL (Medium N° 3 in **Table 1**), further experiments were conducted under the same cultivation conditions, varying the concentrations of glucose and CSL, as described in **Table 2**. After the static cultivation of the bacterium for 10 days, the BC pellicles produced in the surface of the cultivation medium were harvest with the aid of a metal clamp. The mass and the thickness of the BC pellicles were determined using a precision balance and a micrometer, respectively, to compare the quality of the films obtained with the different cultivation media. After selection of the best concentration of glucose and CSL, the BC membranes were purified and characterized in terms of chemical structure, crystallinity, and morphology using XRD and SEM.

**Table 2:** Variations in alternative medium composition formulated with glucose and CSL.

New alternative media	Glucose (g/L)^∗^	CSL (g/L)^∗^
10	20	15
11	20	35
12	40	15
13	40	35
14	15	25
15	45	25
16	30	10
17	30	40
18	30	25

*^∗^Concentration in the alternative medium formulated with 2.7 g/L disodium phosphate and 1.5 g/L citric acid monohydrate.*

### Harvesting and Weighing of BC

After cultivation, the BC membranes were washed with water and soaked in 0.1 M NaOH at 80°C for 2 h to remove bacterial cells possibly attached to the BC pellicles. Then, the pellicles were washed with deionized water several times to warrant the complete remove the alkali, leaving the pellicle at neutral pH. The purified cellulose was dried at 60°C for 12 h until reaching a constant mass. Triplicate experiments were performed for each membrane and mean values were calculated (Wu et al., 2014).

### Scanning Electron Microscopy

For SEM, the dried BC membrane was mounted on a copper stub using double adhesive carbon conductive tape and coated with gold for 30 s (SC-701 Quick Coater, Japan). The SEM photographs were obtained using a scanning electron microscope (550 Superscan, Shimadzu, Japan) operating at 15 kV at room temperature.

### X-ray Diffractrometry

X-ray diffraction (XRD) patterns of all BC membranes were measured using a diffractometer (Bruker D8 Advance Davinci) with Cu Kα radiation. The crystallinity percentage was measured by equation of Segal given below.

(1) $$Crl=\frac{\left(I_{002}-I_{\text{am}}\right)}{I_{002}}\times 100$$

In this equation, where *Crl* expresses the relative degree of crystallinity, *I*_002_ is the maximum intensity (in arbitrary units) of the 002 lattice diffraction, and *I*_am_ is the intensity of diffraction in the same units at 28 = 18°.

### Thermogravimetric Analysis

Thermogravimetric analysis (TGA) measurements were performed using a simultaneous thermal analyzer (STA 6000, PerkinElmer) on samples of about 8 mg. Each sample was scanned over a temperature range from room temperature to 800°C with a heating rate of 10°C/min under nitrogen with a flow rate 50 mL min^-1^ to avoid oxidation. Six randomly selected ground (fine particles) samples were used for the TGA measurements (Kiziltas et al., 2015).

### Tensile Strength Testing of BC

For the films produced in the alternative medium and in the HS medium, tensile strength (N), tensile strength at peak load (MPa), elongation at break (𝜀) (%), and Young’s modulus of elasticity (E) were determined based on Rethwisch and William (2016). The BC sheets (thickness of 0.48 ± 0.0 and 8.7 ± 0.01 mm for dried and hydrated sheets, respectively) were cut into rectangular strips (70 mm × 20 mm). Tensile testing was performed at room temperature at a speed of 1 mm/min and a static load of 0.5 N using a universal testing machine (EMIC DL – 500MF, Brazil) following the ASTM D882 Method for Tensile Properties of Thin Plastic Sheeting. The Bluehill Lite^TM^ software program was used to calculate the stress–strain relationship and modulus of elasticity. A crosshead displacement sensor was used for the deformation measurements.

### Statistical Analysis

In order to verify the existence of differences between the average responses of the treatments, analysis of variance (ANOVA) tests were performed. Before, however, the data that composed each midpoint were preliminarily compared with the help of box plot. The one-way ANOVA test was used, and it was concluded that there were significant differences between the means of the treatments at the significance level of *p* < 0.05.

## Results

### Production of BC in Alternative Media

Bacterial cellulose (BC) pellicles were produced through fermentation at 30°C for 10 days. The pH, temperature, and the ratio of substrate surface area to volume proved to be very important to optimal BC production, since the surface area of the culture medium is inversely proportional to the thickness of the wet pellicle. For the production of membranes with a surface area of 44.17 cm^2^ and a mean thickness of 0.62 ± 0.08 mm, it was necessary to use a volume of culture medium equal to 100 mL and Schott vessels of 500 mL and diameter of 7.50 mm.

The replacement of synthetic compounds with industrial residues in the HS medium enabled the production of BC under static conditions in some of the media tested. The residues used in this study did not undergo treatment prior to use. However, the jeans laundry effluent was sterilized to avoid contamination of the medium. After 10 days of fermentation, the media containing acetylated glucose (Media 1 and 9) and laundry effluent (Media 5, 6, 7, and 8) did not produce BC. Regarding the acetylated glucose, it was observed that it had a low solubility in the medium and after autoclaving the degradation of this compound occurred.

The objective of using the jeans laundry effluent was to produce an already colored pellicle for industrial application, thus avoiding the dyeing step, which represents a high environmental impact. According to the State Agency of Environment and Water Resources of the State of Pernambuco, Brazil, the volume of wastewater generated in the laundries is close to 55985.25 m^3^/month (Lima et al., 2005). Another reason would be to give the effluent generated in the laundries an applicability, avoiding their disposal in the water bodies. The visual observation of the effluent allowed to detect textile fibers dispersed in the liquid. These fibers could be entangled together with the BC fibers produced in the medium, thus forming a biocomposite. Although the sterilized effluent has maintained its initial color and pH, the results obtained with the alternative media formulated in its presence were not satisfactory. Probably, the artificial dyes present inhibited the production of BC by the bacterium, as also reported in the literature (Bertazzoli and Pelegrini, 2002; Kunz et al., 2002; Pinheiro et al., 2004).

The hydrated BC pellicles were obtained in the presence of glucose, molasses, and CSL (Media 2, 3, and 4). Medium N° 3 – formulated with glucose as the carbon source and CSL as the nitrogen source to replace yeast extract and peptone – was selected for further study based on the yields in hydrated (367.63 ± 0.5) and dry (5.80 ± 0.5) mass shown in **Table 3**.

**Table 3:** Hydrated and dry mass of BC pellicles produced in alternative media compared to HS medium.

Culture medium	HS	1	2	3	4	5	6	7	8	9
Hydrated mass (g/L)	518.10 ± 0.6	–	35.27 ± 0.8	367.63 ± 0.5	105.8 ± 0.7	–	–	–	–	–
Dry mass (g/L)	8.00 ± 0.9	–	0.30 ± 0.6	5.80 ± 0.5	0.90 ± 0.6	–	–	–	–	–

*Results are expressed as mean ± pure error.*

The availability of the carbon and nitrogen sources in the culture medium favors the production of BC, especially noting that the yields of BC were dependent on the carbon source. Thus, the media which presented toxic compounds as dyes, i.e., the media containing the laundry effluent, did not allow the production of BC, as there was an inhibition or destruction of the *G. hansenii* cells. Acetylation of glucose, on the other hand, caused physical and chemical modifications to glucose, making the availability of the substrate difficult to the microorganism. The substitution of 100% of glucose by sugarcane molasses, in turn, resulted in a minimal production of BC. The substitution of the synthetic nitrogen source by the CSL residue allowed a 25% reduction of the glucose favoring the production of films of excellent quality.

Further fermentations were conducted varying the concentrations of glucose and CSL, as shown in **Table 4**. The alternative modified HS medium denominated N° 14, containing 15 g/L glucose and 25 g/L CSL, achieved the highest yields in terms of dry (9.63 ± 0.9 g/L) and hydrated (381.10 ± 0.8 g/L) mass. The hydrated pellicle produced with this alternative medium corresponded to 73% of the production achieved with the standard HS medium. The result obtained in this medium is satisfactory especially considering the results described for other media formulated with industrial residues as it will be described below. Statistical analysis indicated significant differences (*p* < 0.05) in the yields of the pellicles showed in **Tables 3 and 4**.

**Table 4:** Hydrated and dry mass obtained from alternative media formulated with glucose and CSL.

Alternative media Identification	Hydrated mass (g/L)	Dry mass (g/L)
10	381.00 ± 0.8	9.30 ± 0.5
11	302.10 ± 0.7	8.80 ± 0.8
12	212.40 ± 0.5	3.55 ± 0.4
13	284.40 ± 0.6	5.34 ± 0.8
14	381.10 ± 0.8	9.63 ± 0.9
15	138.60 ± 0.7	4.60 ± 0.8
16	162.50 ± 0.5	6.54 ± 0.3
17	176.50 ± 0.8	3.79 ± 0.9
18	281.20 ± 0.4	4.03 ± 0.6

*Results are expressed as mean ± pure error.*

**FIGURE 1:**
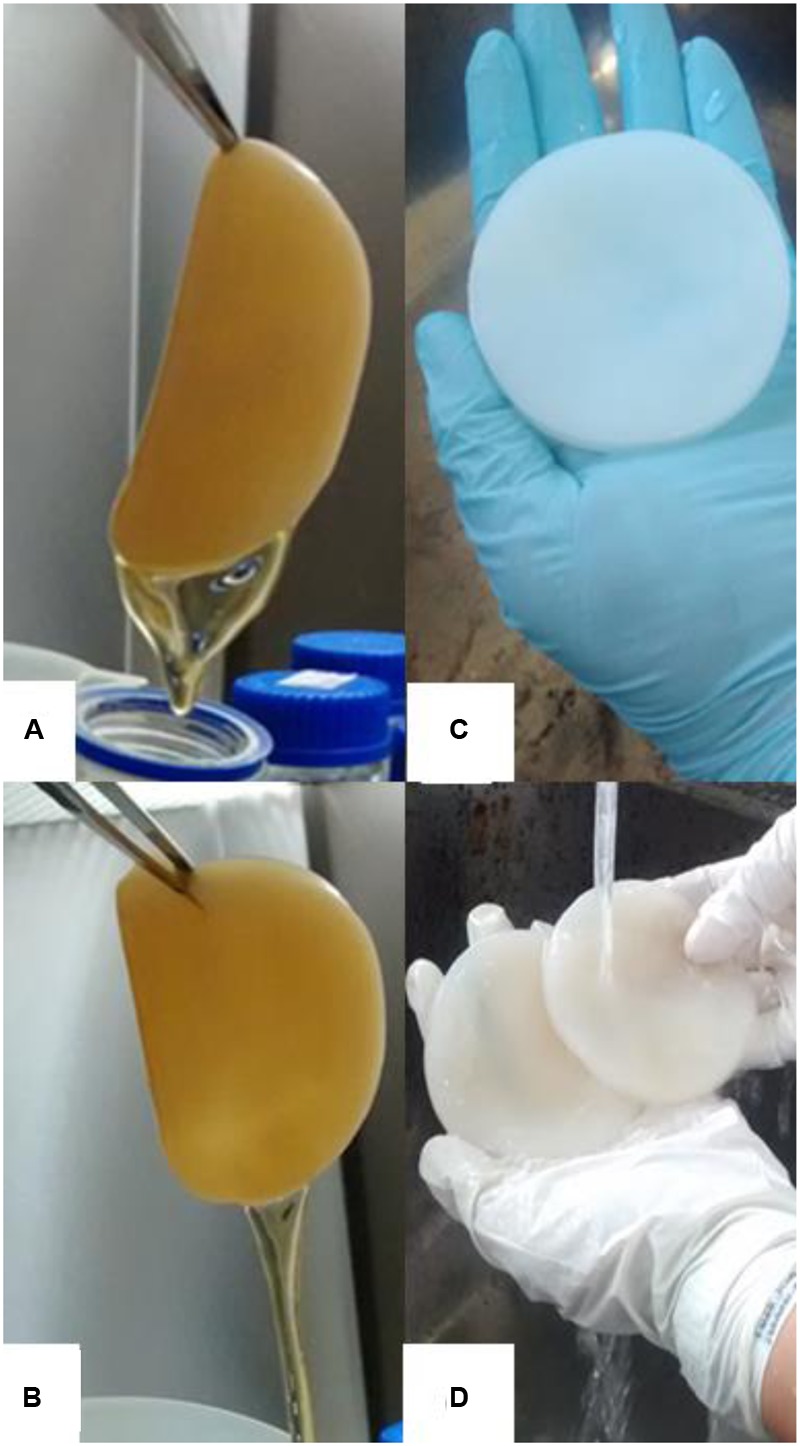
Macrograph of bacterial cellulose (BC) pellicles composed by nanofibrils and water produced after 10 days of static cultivation in Hestrin–Schramm (HS) medium **(A)** and in alternative medium **(B)**. Pellicles produced before **(A,B)** and after NaOH treatment to remove *G. hansenii* biomass **(C,D)**.

The pellicles produced in the alternative medium exhibited similar thickness as well as visual and tactile texture to the control (HS medium) and demonstrated a yield 3.53% higher than that achieved previously (**Table 3**), with a 25% reduction in the carbon source and 100% replacement of the nitrogen source with CSL in comparison with the HS medium (**Figures 1A–D**). As shown in **Figure 1A**, a light brownish pellicle was obtained with the alternative culture medium. After the pellicles were treated in NaOH to remove the *G. hansenii* biomass, purified white pellicles were obtained (**Figure 1C**). Membrane thickness was estimated to be 0.2–0.5 mm. The color intensity of the pellicles produced in media containing CSL was slightly higher than that produced with the HS medium.

### Scanning Electron Microscopy (SEM)

**FIGURE 2:**
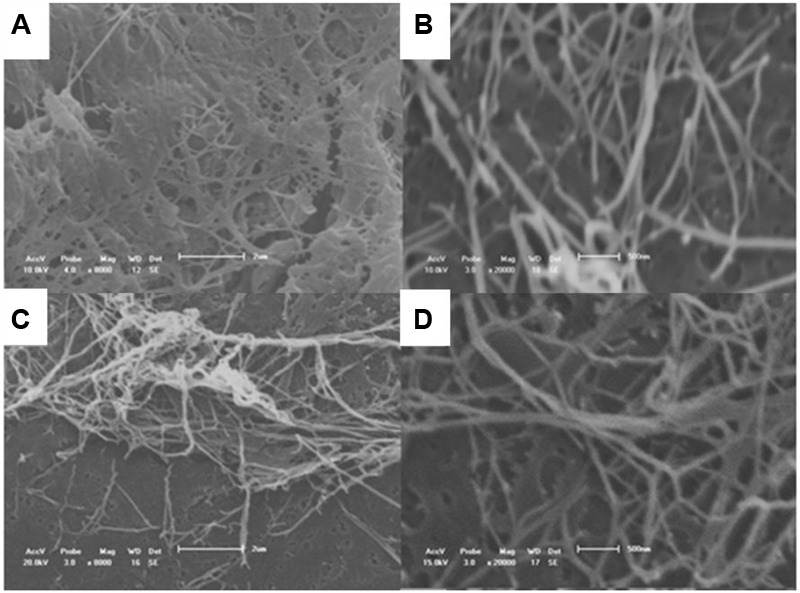
SEM images of BC pellicles obtained after 10 days of fermentation in **(A)** Hestrin–Schramm (HS) medium (2% glucose, 0.5% yeast extract, 0.5% peptone, 0.27% Na_2_HPO_4_, and 1.5% citric acid) and **(B)** alternative medium formulated with 1.5% glucose, 2.5% CSL, 0.27% Na_2_HPO_4_, and 1.5% citric acid. Magnification: 8,000 **(A,B)** and 20,000 times **(C,D)**.

**Figure 2** displays the micrographs of the surface of the BC produced with the alternative medium under the optimal conditions selected in this study and the standard HS medium, showing the dispersion of the fibers and interfacial adhesion. It is notable that the BC pellicles synthesized with the alternative medium were stable. The compact cellulose network structure consisted of a random assembly of fibrils. Both purified BC pellicles exhibited a reticulated structure consisting of ultrafine nanofibrils and no significant difference in size, as illustrated in the SEM images. Greater microfibril and nanofibril production was achieved when CSL was used in place of synthetic compounds.

The BC produced in the modified HS medium in the present study exhibited fine cellulose fibers, which formed a porous three-dimensional network structure. It is important to note that the application of heat for drying the films was instrumental in the concentration of the network structure. According to Santos et al. (2015), high temperatures shorten the distance between the layers of BC and assist in the evaluation of porosity. The visualization of the fiber network magnified 8,000 and 20,000× times demonstrated a smooth surface of the fibers produced with both media.

### Thermogravimetric Analysis

**FIGURE 3:**
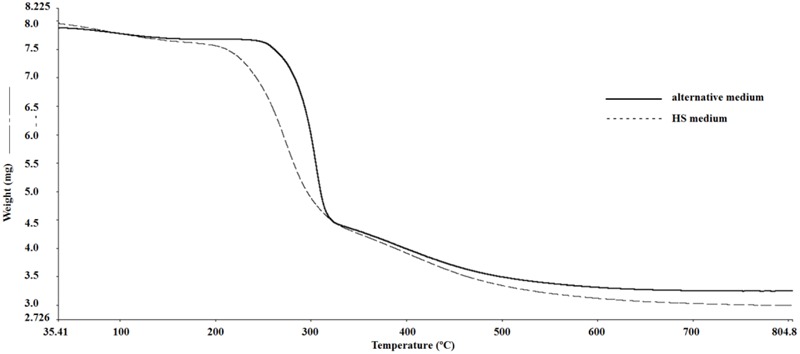
TG curves of BC pellicles obtained after 10 days of fermentation in Hestrin–Schramm (HS) medium (2% glucose, 0.5% yeast extract, 0.5% peptone, 0.27% Na_2_HPO_4_, and 1.5% citric acid) and obtained in the alternative medium formulated with 1.5% glucose, 2.5% CSL, 0.27% Na_2_HPO_4_, and 1.5% citric acid.

**Figure 3** shows the thermogravimetric degradation curves of the dried pellicles in percentage of the mass of the original sample as a function of temperature. During the initial thermal treatment, the samples exhibited slight mass loss beginning with room temperature up to 200°C due to the loss of water. A second, significant mass loss occurred around 300°C, which may be attributed to the decomposition of the samples. This event also may be associated with the degradation of cellulose, including the depolymerization, dehydration, and decomposition of glucose units.

The BC produced in the alternative medium (**Figure 3**) did not decompose before 250°C, while the BC produced in the HS medium exhibited a 2.6% decomposition rate at 195°C. Above 700°C, mass loss rates were low and the charred ashes corresponded to approximately 37% for the BC produced with the HS medium and 42% for that produced with the alternative modified HS medium. The agro-industrial residue conferred more thermal stability to the BC pellicle produced in the alternative medium than that produced in the standard HS medium. The use of an industrial waste by-product in the production of BC can help minimize environmental impacts and reduce BC production costs, thereby aggregating economic value to the pellicle.

Maximum decomposition temperature is a criterion of thermal stability. Thermal degradation is affected by structural variables, such as molecular mass, crystallinity, and the arrangement of the fibers (Vazquez et al., 2013). Maximum decomposition occurred at 310°C for the BC produced with the alternative medium and 290°C for that produced with the standard HS medium. These results indicate that greater thermal stability of the BC produced in the alternative medium.

### X-ray Diffraction (XRD) Analyses

**FIGURE 4:**
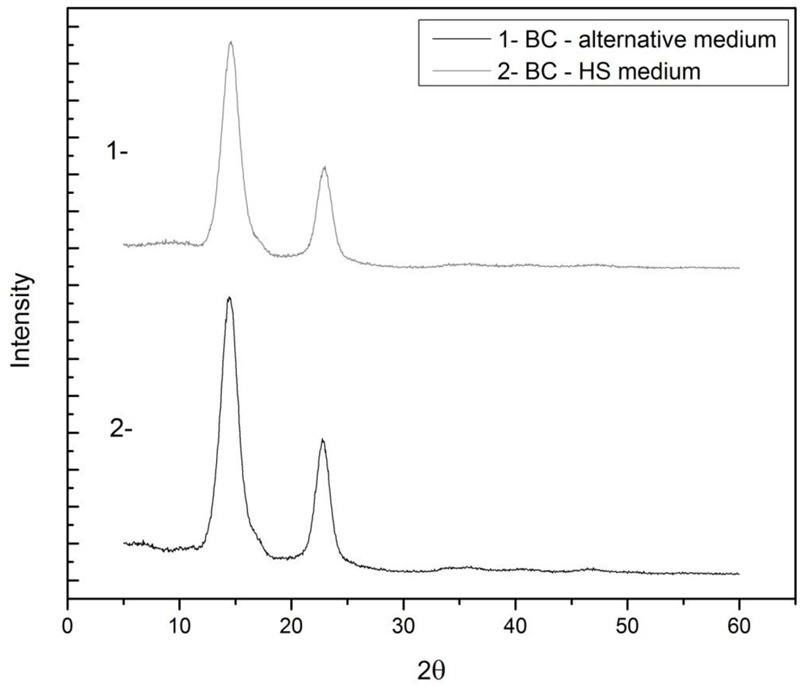
X-ray diffraction diagrams of BC dried pellicles obtained after 10 days of fermentation in Hestrin–Schramm (HS) medium (2% glucose, 0.5% yeast extract, 0.5% peptone, 0.27% Na_2_HPO_4_, and 1.5% citric acid) and in the alternative medium formulated with 1.5% glucose, 2.5% CSL, 0.27% Na_2_HPO_4_, and 1.5% citric acid.

X-ray diffraction (XRD) was used to evaluate the crystalline structure as well as the change in crystallinity of the BC produced with the two media. As shown in **Figure 4**, the peaks at 2𝜃 angles of 14.422 and 22.996° for the BC produced with the alternative medium and at 2𝜃 angles of 14.422 and 22.747° for the BC produced with the standard HS medium corresponded to the (-1 1 0) and (2 0 0) crystal planes, respectively, demonstrating that the products obtained from both media were cellulose I.

The crystallinity index was calculated based on peak intensity data determined using the Segal method (Cheng et al., 2009). The crystallinity index for the BC produced with the alternative medium (84.9%) was practically the same of that obtained with the HS medium (86.1%). These results show that the composition of the medium did not affect the crystalline morphology of the polymers and the physical characteristics of the membranes were maintained. Moreover, crystallinity has an inversely proportional relationship with the porosity of the cellulose surface, as a lower throughput denotes greater pore size in the cellulose structure. The fact that the pores increase in size allows a greater amount of water molecules to penetrate and be absorbed by the membrane, demonstrating an increase in the degree of hydrophilicity.

Moreover, crystallinity has an inversely proportional relationship with the porosity of the cellulose surface, i.e., the larger the size of the crystals and the proximity between them, the smaller the number of pores and the lower the hydrophilicity of the polymer. The fact that the pores increase in size allows a greater amount of water molecules to penetrate and be absorbed by the membrane, demonstrating an increase in the degree of hydrophilicity. The images presented in **Figures 2A,C** show a higher concentration of fibers and nanofibers of BC in the pellicle produced in the alternative medium, justifying the difference in mass between the films, even though they have very close *Crl* values.

Another important aspect concerns the type of membrane purification. Heat treatment with NaOH facilitates the removal of certain metabolites and increases viscosity, promoting purification and the elimination of low molecular mass cellulose, which confers better characteristics to the biomaterial.

### Tensile Strength of BC

**FIGURE 5:**
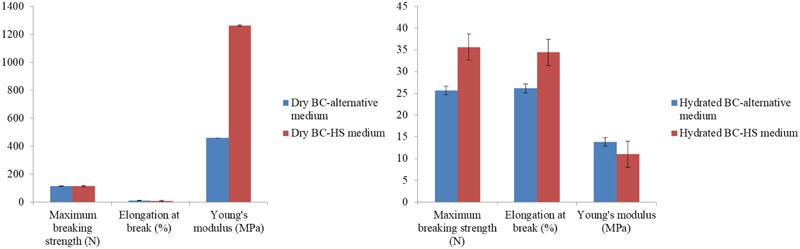
Mechanical properties of dry and hydrated BC pellicles produced with Hestrin–Schramm (HS) medium (2% glucose, 0.5% yeast extract, 0.5% peptone, 0.27% Na_2_HPO_4_, and 1.5% citric acid) and produced with the alternative medium formulated with 1.5% glucose, 2.5% CSL, 0.27% Na_2_HPO_4_, and 1.5% citric acid.

**Figure 5** shows the results of the tensile assay used to characterize the mechanical properties of the hydrated and dry pellicles produced with the alternative medium and with the HS medium. The results were similar between the pellicles. The main difference was observed in the Young’s modulus of the dry BC pellicle produced in the HS medium, which was superior when compared to the pellicle obtained in the alternative medium. The hydrated pellicles obtained in the alternative medium had mean tensile strength of 2.29 ± 0.94 MPa when subjected to an average force of 25.67 N while the dry pellicle had mean tensile strength of 48.17 ± 15.38 MPa when the average maximum force was 115.59 N at the breaking point. Considerable variation in the results is found when hydrated and dry samples of the same polymer are submitted to mechanical tests. According to Almeida et al. (2013) and Gao et al. (2016), water acts as a plasticizer. The hydrated pellicle from the alternative medium had greater elongation at break, stretching 56.60% more than the dry pellicle.

The water and waste by-products in the culture medium acted as plasticizers and altered the mechanical performance of the biomaterial. The water in hydrated pellicles decreases the number of hydrogen bonds between molecules and reduces intermolecular forces, as demonstrated by the results of the mechanical tests, in which the hydrated pellicle exhibited greater elongation and lower tensile strength than the dry pellicle.

Considerable plastic deformation of the pellicle was observed before rupture in the form of an irreversible ductile fracture of the biopolymer. The propagation of cracks happened slowly and accompanied by necking during the tensile test.

Regarding deformation, the hydrated and dry pellicles did not return to the natural (non-deformed) state. The increase in delamination or crack propagation occurs in dry pellicles faster than hydrated pellicles due to the presence of water and residues from the culture medium, demonstrating that the hydrogel does not have elasticity even when subjected to low stress. Young’s modulus of elasticity (E) is an intrinsic property used as a mechanical parameter directly related to polymer stiffness (Almeida et al., 2013). The amount of water held between BC fibers is a determining factor of the greater deformation of hydrated pellicles than dry pellicles, since water acts as a plasticizer and its absence makes biomaterial brittle and hard. In the present study, the mean modulus of elasticity of the dry pellicle was 96.98% higher than that of the hydrated pellicle, which demonstrates the mechanical strength of the biomaterial.

**FIGURE 6:**
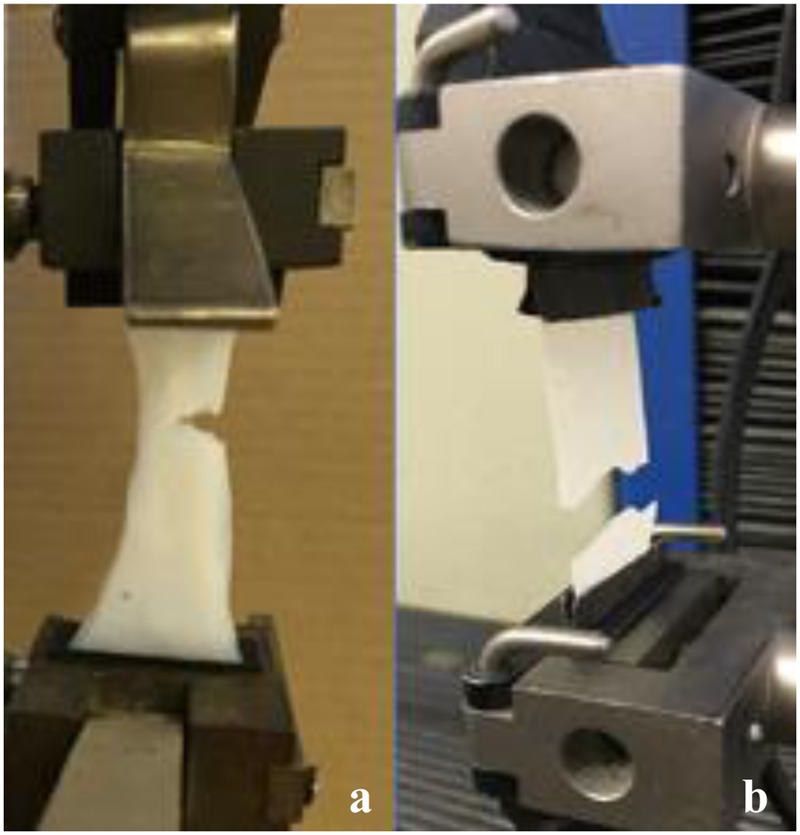
Tensile assay of hydrated **(a)** and dry **(b)** pellicles of BC obtained in alternative medium formulated with 1.5% glucose, 2.5% CSL, 0.27% Na_2_HPO_4_, and 1.5% citric acid until complete rupture of film.

Macroscopically, it was difficult to identify the point of rupture and the alignment behavior of the fibers of the BC pellicle obtained from the alternative medium when submitted to stress (**Figure 6**). The hydrated and dry pellicles initially broke transversely at a point, but remained whole with the increase in load during the tear process until the complete rupture of the film. This occurred because the pellicles had microfibers and nanofibers that were disorderly arranged as a non-woven fibrous network that contained a significant amount of water and residues from the culture medium.

## Discussion

In the present study, BC nanofibers were synthesized using *G. hansenii* cultivated in different culture media. Glucose, acetylated glucose, and sugarcane molasses were tested as the carbon sources and CSL, yeast extract, and peptone were tested as nitrogen sources. It is important to note that CSL efficiently replaced yeast extract and peptone. Since CSL contains 21–45% proteins, 20–26% lactic acid, approximately 8% ash (Ca^2+^, Mg^2+^, K^+^, etc.), approximately 3% carbohydrates and a low fat content (0.9–1.2%), this by-product of the corn wet-milling process constitutes a low-cost substrate that is very rich in nutrients for the production of various metabolites by microorganisms (Santos et al., 2016). The addition of 8% CSL to utilized coffee cherry husk (CCH) allowed a 47% increase in BC production, as described by Rani and Appaiah (2013). Jung et al. (2010b) obtained a maximum BC yield (12 g/L on a dry basis) under the addition of CSL. A mixture of 4% of carbon sources (glucose: fructose, 1: 3) with 10% CSL as the nitrogen source provided excellent results and exhibited a production of BC by *Acetobacter xylinum* KJ-1 of 7.2 g/L, about 3 times more than the obtained with the HS medium (Son et al., 2002).

Some authors report that the structure of cellulose is not affected by changing the carbon or nitrogen source (Nguyen et al., 2010), but others have reported differences. El-Saied et al. (2007) found that a medium containing CSL and molasses resulted in a higher degree of crystallization in comparison with the use of carbon and nitrogen sources such as glucose, mannitol, yeast extract, and peptone, whereas Jung et al. (2010a) found a decrease in crystallinity in a molasses medium when compared to a complex control medium.

In a previous study, cellulose production by *G. intermedius* SNT-1 under static conditions was compared using different carbon sources, including molasses. A yield of 12.6 g/L (on a dry basis) of BC was obtained using 45.8 g/L of diluted (1:4 v/v) molasses after heat pre-treatment with H_2_SO_4_. Cellulose pellicles yields were comparable using CSL or yeast extract as the nitrogen sources along with the heat pre-treated molasses (Tyagi and Suresh, 2016).

Organic nitrogen sources are generally preferred for effective BC production. Hungund and Gupta (2010) found that beef extract was the best nitrogen source for BC production by *G. persimmonis* GH-2. The highest yield (5.89 g/L on a dry basis) was achieved with 0.6% beef extract in the medium, followed by casein hydrolysate, peptone, malt extract, ammonium chloride, potassium nitrate, ammonium sulfate, sodium nitrate, and ammonium nitrate. Mohammadkazemi et al. (2015) found that yeast extract and peptone were indispensable nitrogen sources for cell growth and BC production using *G. xylinus* strain PTCC 1734 in different media.

Kiziltas et al. (2015) studied the production of BC by *G. xylinum*. Maximum production (0.15 g/L) occurred at pH 8 and 28°C using a medium formulated with a residual material originating from pulp mills and lignocellulosic biorefineries. Lin et al. (2014) described the production of BC by *G. hansenii* CGMCC 3917 with a two-step pre-treatment process, in which a sugar concentration of 3% waste beer yeast hydrolysates led to the highest yield (7.02 g/L on a dry basis). Gomes et al. (2013) produced BC by *G. sacchari* cultivated in dry olive mill residue. Without the addition of any nutrients, production was 0.85 g/L for after 96 h of incubation, corresponding 34% of the production achieved with the standard HS culture medium (around 2.5 g/L), whereas the supplementation of nitrogen and phosphate sources increased BC production to 43.2%.

Regarding the decomposition temperature, although our results indicated the greater thermal stability of the BC produced in the alternative medium, Kiziltas et al. (2015) reported that BC from a medium with a hot water extract containing wood sugars had lower thermal stability compared to BC from the HS medium and El-Saied et al. (2007) found that plant cellulose exhibited a much slower rate of mass loss, with peaks at 342 and 501°C.

A considerable plastic deformation of the pellicle was observed before rupture. According to Gao et al. (2016), the propagation of cracks in polymers is primarily associated with crazing at the crack tip during the tensile test. A decrease in strength occurs, beginning with a fracture followed by cracks, which are stabilized for a few seconds by the BC microfibrils and nanofibrils until the onset of the rupture (Almeida et al., 2013). The movement of water contributes to the viscous behavior of the BC hydrogel subjected to tensile tests (Frensemeier et al., 2010). A BC hydrogel studied by Gao et al. (2016) changed with the increase in the stress level, including changes in the arrangement of the fibers, their local volume fraction, porosity, and the interaction between the fibers and water, resulting in the stiffening of the material (Astley et al., 2003; Frensemeier et al., 2010). When a tensile load is applied to a sample, the fibers attempt to rearrange and nodes are formed in the direction of the load, while the interstitial water resists the movement of the fibers due to its low compressibility and is squeezed out from the fibrous network during the lateral shrinkage process. Indeed, fiber stretching plays a major role in the deformation of BC pellicles (Kowalska-Ludwicka et al., 2013). According to Gao et al. (2016), the behavior of BC hydrogels varies with the increase in stress and is positively correlated with stiffness, demonstrating instantaneous shear at the linear stress creep.

The combination of qualitative microstructural observations and quantitative force–displacement relations enabled the identification of the main deformation mechanisms as well as the confirmation of the inelastic behavior of the BC hydrogel under a loading–unloading–reloading protocol. Gao et al. (2015) identified six main deformation mechanisms when BC hydrogels are submitted to cyclic tension: (1) the reorientation of fibers; (2) the interaction of fibers; (3) the deflection of molecule chains; (4) the interaction between entanglements; (5) the elongation of the covalent bond of molecule chains; and (6) the prevention of fiber movement by interstitial water.

Shao et al. (2015) found cellulose microfibrils on fracture surfaces of a cellulose nanofiber-modified composite. Energy absorption mechanisms, such as fiber debonding, plastic void growth, matrix deformation, and fiber-bridging, can strengthen the matrix to resist the propagation of cracks. The author identified the accumulation of internal fatigue damage in the fabric composite with the increase in the loading cycles, such as fiber debonding, transverse cracks, meta-delamination at the crossover points between warp and weft, interlaminar delamination, and breakage. The addition of cellulose nanofiber between 0.3 and 0.8% to the matrix extended the fatigue life by 10–30 times at different applied stress compared to unmodified composite.

This production of BC pellicles with a low-cost medium was successfully reached. The cost of the alternative medium was calculated, presenting an economy of 57.94% in relation to the HS medium. The results demonstrated the potential of using CSL in the production of BC. CSL was used as a nitrogen source to replace the synthetic nitrogen sources in the standard medium and also favored a 25% reduction in the concentration of glucose. With these substitutions, a 57.94% reduction was achieved in the cost of producing BC with *G. hansenii* in a modified HS medium, with 73.55% productivity for the hydrated pellicle, compared to production obtained with the standard HS medium. Although the yield was lower than that reported for the reference HS medium, the results are promising due to the fact that a low-cost waste by-product was used. So, this investigation shows good perspectives for future valorization studies of more residues by producing a biomaterial with unique properties such as BC. According to Huang et al. (2014) the research around fermentation for enhancing productivity should be concentrated on further.

The mechanical, thermal, and physical properties of the BC pellicles produced with the alternative medium indicate possible applications of this biomaterial in the textile, medical, and food industries, as described in the literature (Petersen and Gatenholm, 2011; Torres et al., 2012; Ashjaran et al., 2013; Shi et al., 2014; Tang et al., 2014; Jorfi and Foster, 2015; Costa et al., 2017). Uses in the development of textile products will be tested in the next step.

## Conclusion

This paper presented an experimental study on the production of BC pellicles with an alternative, low-cost medium containing industrial waste. A description of the main characteristics of this biomaterial was also performed for future applications in the textile field. Considering the results obtained for elasticity and tensile strength in the tests performed, the future use of the BC for the textile and clothing area can give durability and comfort to the wearable artifacts during the use and maintenance of the product. As shown, the thermoresistance of the BC makes it possible to perform dyeing, which generally requires high temperatures. Aspects related to the production and consumption of eco-friendly products are intangible values that are associated with the presented properties. The arrangement of the microfibrils/nanofibrils and the amount of water were major factors during the determination of the mechanical properties of the BC, such as tensile strength and deformation of the biomaterial. The drying of the material exerted a considerable influence on mechanical strength, whereas the presence of water served as a plasticizer, increasing the elasticity of biocellulose produced in the modified HS medium and provided a dispersion of the fibers and nanofibers of BC in the wet film. Besides being economical, CSL as a residual raw material for the production of BC helps minimize the environmental impact resulting from the disposal of this industrial waste and can help reduce deforestation caused by the use of vegetal cellulose. It can therefore be concluded that the bacterial production of cellulose is a cleaner option. Although plant cellulose cannot be entirely replaced by BC due to the low yield and long time required for mass production, BC may be a suitable option for the generation of different products and composites. Further studies will be conducted for the development of numerical analysis tools to identify properties of the pellicle for applications in the textile industry. Changes in the concentration of the BC per mm^2^ and/or the development of a physical mechanism using a reinforcing material for the reorientation of the fibers and increased hydrophobicity of the pellicle will be considered in future studies with the aim of increasing the mechanical strength of the biomaterial.

## Author Contributions

All authors contributed to this work. LS conceived and designed the experiments. AC, FA, and GV performed the experiments. LS and GV analyzed the data and contributed to analysis tools. LS, AC, and GV wrote the paper.

## Conflict of Interest Statement

The authors declare that the research was conducted in the absence of any commercial or financial relationships that could be construed as a potential conflict of interest. The reviewer SR and handling Editor declared their shared affiliation.

## References

[B1] Almeida L. R. Martins A. R. Fernandes E. M. Oliveira M. B. Correlo V. M. Pashkuleva I. (2013). New biotextiles for tissue engineering: development, characterization and in vitro cellular viability. *Acta Biomater.* 9 8167–8181. 10.1016/j.actbio.2013.05.019 23727248

[B2] Ashjaran A. Yazdanshenas M. E. Rashidi A. Khajavi R. Rezaee A. (2013). Overview of bio nanofabric from bacterial cellulose. *J. Text. Inst.* 104 121–131. 10.1080/00405000.2012.703796

[B3] Astley O. M. Chanliaud E. Donald A. M. Gidley M. J. (2003). Tensile deformation of bacterial cellulose composites. *Int. J. Biol. Macromol.* 32 28–35. 10.1016/S0141-8130(03)00022-9 12719129

[B4] Bertazzoli R. Pelegrini R. (2002). Descoloração e degradação de poluentes orgânicos em soluções aquosas do processo fotoeletroquímico. *Quím. Nova* 25 477–482. 10.1590/S0100-40422002000300022

[B5] Borzani W. Souza S. J. (1995). Mechanism of the film thickness increasing during the bacterial production of cellulose on non-agitated liquid media. *Biotechnol. Lett.* 17 1271–1272. 10.1007/BF00128400

[B6] Brown A. J. (1886). An acetic ferment which forms cellulose. *Chem. Soc.* 49 432–439.

[B7] Budhiono A. Rosidi B. Taher H. Iguchi M. (1999). Kinetic aspects of bacterial cellulose formation in nata-de-coco culture system. *Carbohydr. Polym.* 40 137–143. 10.1016/S0144-8617(99)00050-8

[B8] Çakar F. Özer I. Aytekin A. Ö. Şahin F. (2014). Improvement production of bacterial cellulose by semi-continuous process in molasses medium. *Carbohydr. Polym.* 106 7–13. 10.1016/j.carbpol.2014.01.103 24721044

[B9] Cavka A. Guo X. Tang S. J. Winestrand S. Jönsson L. J. Hong F. (2013). Production of bacterial cellulose and enzyme from waste fiber sludge. *Biotechnol. Biofuels* 6 1–10. 10.1186/1754-6834-6-25 23414733 PMC3610104

[B10] Chawla P. R. Bajaj I. B. Survase S. A. Singhal R. S. (2009). Microbial cellulose: fermentative production and applications. *Food Technol. Biotechnol.* 47 107–124.

[B11] Cheng K. C. Catchmark J. M. Demirci A. (2009). Effect of different additives on bacterial cellulose production by *Acetobacter xylinum* and analysis of material property. *Cellulose* 16 1033–1045. 10.1007/s10570-009-9346-5

[B12] Costa A. F. S. Rocha M. A. V. Sarubbo L. A. (2017). Bacterial cellulose: an ecofriendly biotextile. *Int. J. Text. Fashion Technol.* 7 11–26.

[B13] Czaja W. Krystynowicz A. Bielecki S. Brown R. M. Jr. (2006). Microbial cellulose the natural power to heal wounds. *J. Biomater.* 27 145–151. 10.1016/j.biomaterials.2005.07.035 16099034

[B14] Czaja W. K. Young D. J. Kawecki M. Brown R. M. Jr. (2007). The future prospects of microbial cellulose in biomedical applications. *Biomacromolecules* 8 1–12. 10.1021/bm060620d 17206781

[B15] Donini Í. A. N. Salvi D. T. B. Fukumoto F. K. Lustri W. R. Barud H. S. Marchetto R. (2010). Biosynthesis and recent advances in production of bacterial cellulose. *Eclética Quím.* 35 165–178. 10.1590/S0100-46702010000400021

[B16] El-Saied H. Basta A. H. Gobran R. H. (2007). Research progress in friendly environmental technology for the production of cellulose products (Bacterial cellulose and its application). *Polym. Plast. Technol. Eng.* 43 797–820. 10.1081/PPT-120038065

[B17] Esa F. Tasirin S. M. Rahman N. A. (2014). Overview of bacterial cellulose production and application. *Agric. Agric. Sci. Proc.* 2 113–119. 10.1016/j.aaspro.2014.11.017

[B18] Fernandes S. C. M. Oliveira L. Freire C. S. R. Silvestre A. J. D. Pascoal Neto C. Gandini A. (2009). Novel transparent nanocomposite films based on chitosan and bacterial cellulose. *Green Chem.* 1 2023–2029. 10.1039/b919112g

[B19] Frensemeier M. Koplin C. Jaeger R. Kramer F. Klemm D. (2010). Mechanical properties of bacterially synthesized nanocellulose hydrogels. *Macromol. Symp.* 294 38–44. 10.1002/masy.200900030

[B20] Gao X. Shi Z. Kuśmierczyk P. Liu C. Yang G. Sevostianov I. (2016). Time-dependent rheological behaviour of bacterial cellulose hydrogel. *Mater. Sci. Eng.* 58 153–159. 10.1016/j.msec.2015.08.019 26478298

[B21] Gao X. Shi Z. Liu C. Yang G. Sevostianov I. Silberschmidt V. V. (2015). Inelastic behaviour of bacterial cellulose hydrogel: in aqua cyclic tests. *J. Polym. Test.* 44 82–92. 10.1016/j.polymertesting.2015.03.021

[B22] Gayathry G. Gopalaswamy G. (2014). Production and characterization of microbial cellulosic fibre from *Acetobacter xylinum*. *Indian J. Fibre Text. Res.* 39 93–96.

[B23] Goelzer F. Faria-Tischer P. C. S. Vitorino J. C. Sierakowiski M. R. Tischer C. A. (2009). Production and characterization of nanospheres of bacterial cellulose from *Acetobacter xylinus* from processed rice bark. *J. Mater. Sci. Eng. C* 29 546–551. 10.1016/j.msec.2008.10.013

[B24] Gomes F. P. Silva N. H. C. S. Trovatti E. Serafim L. S. Duarte M. F. Silvestre A. J. D. (2013). Production of bacterial cellulose by *Gluconacetobacter sacchari* using dry olive mill residue. *J. Biomass Bioenergy* 55 205–211. 10.1016/j.biombioe.2013.02.004

[B25] Hestrin S. Schramm M. (1954). Synthesis of cellulose by *Acetobacter xylinum*. Preparation of freeze-dried cells capable of polymerizing glucose to cellulose. *Biochem. J.* 58 345–352. 10.1042/bj0580345 13208601 PMC1269899

[B26] Hong F. Qiu K. (2008). An alternative carbon source from konjac powder for enhancing production of bacterial cellulose in static cultures by a model strain *Acetobacter aceti subsp. xylinus* ATCC 23770. *Carbohydr. Polym.* 72 545–549. 10.1016/j.carbpol.2007.09.015

[B27] Huang Y. Zhu C. Yang J. Nie Y. Chen C. (2014). Recent advances in bacterial cellulose. *Cellulose* 21 1–30. 10.1007/s10570-013-0088-z

[B28] Hungund B. S. Gupta S. G. (2010). Improved production of bacterial cellulose from *Gluconacetobacter persimmonis* GH-2. *J. Microb. Biochem. Technol.* 2 127–133. 10.4172/1948-5948.1000037

[B29] Iguchi M. Yamanaka S. Budhiono A. (2000). Bacterial cellulose e a masterpiece of nature’s arts. *J. Mater. Sci.* 35 261–270. 10.1023/A:1004775229149

[B30] Jeihanipour A. Taherzadeh M. (2009). Ethanol production from cotton-based waste textiles. *Bioresour. Technol.* 100 1007–1010. 10.1016/j.biortech.2008.07.020 18723342

[B31] Jorfi M. Foster E. J. (2015). Recent advances in nanocellulose for biomedical applications. *J. Appl. Polym. Sci.* 41719 1–19. 10.1002/APP.41719

[B32] Jung H. I. Jeong J. H. Lee O. M. Park G. T. Kim K. K. Park H. C. (2010a). Influence of glycerol on production and structural-physical properties of cellulose from *Acetobacter sp*. V6 cultured in shake flasks. *Bioresour. Technol.* 101 3602–3608. 10.1016/j.biortech.2009.12.111 20080401

[B33] Jung H. I. Lee O.-M. Jeong J.-H. Jeon Y.-D. Park K.-H. Kim H.-S. (2010b). Production and characterization of cellulose by *Acetobacter* sp. V6 using a cost-effective molasses–corn steep liquor medium. *Appl. Biochem. Biotechnol.* 162 486–497. 10.1007/s12010-009-8759-9 19730823

[B34] Kiziltas E. E. Kiziltas A. Gardner D. J. (2015). Synthesis of bacterial cellulose using hot water extracted wood sugars. *Carbohydr. Polym.* 124 131–138. 25839803 10.1016/j.carbpol.2015.01.036

[B35] Klemm D. Schumann D. Udhardt U. Marsch S. (2001). Bacterial synthesized cellulose - artificial blood vessels for microsurgery. *Prog. Polym. Sci.* 26 1561–1603. 10.1016/S0079-6700(01)00021-1

[B36] Kongruang S. (2008). Bacterial cellulose production by *Acetobacter xylinum* strains from agricultural waste products. *Appl. Biochem. Biotechnol.* 148 245–256. 10.1007/s12010-007-8119-6 18418756

[B37] Kowalska-Ludwicka K. Cala J. Grobelski B. Sygut D. Jesionek-Kupnicka D. Kolodziejczyk M. (2013). Modified bacterial cellulose tubes for regeneration of damaged peripheral nerves. *Arch. Med. Sci.* 9 527–534. 10.5114/aoms.2013.33433 23847677 PMC3701969

[B38] Kunz A. Peralta-Zamora P. Moraes S. G. Durán N. (2002). Novas tendências no tratamento de efluentes industriais. *Quím. Nova* 25 78–82. 10.1590/S0100-40422002000100014

[B39] Li Z. Wang L. Hua J. Jia S. Zhang J. Liu H. (2015). Production of nano bacterial cellulose from waste water of candied jujube-processing industry using *Acetobacter xylinum*. *Carbohydr. Polym.* 120 115–119. 10.1016/j.carbpol.2014.11.061 25662694

[B40] Lima G. Lucena J. C. Souza R. T. Barros C. R. Rezende R. B. (2005). *Diagnóstico Ambiental das Lavanderias de Toritama - PE. CPRH - Agência Estadual de Meio Ambiente e Recursos Hídricos*. Available at: http://www.cprh.pe.gov.br/downloads/toritama.pdf

[B41] Lin D. Lopez-Sanchez P. Li R. Li Z. (2014). Production of bacterial cellulose by *Gluconacetobacter hansenii* CGMCC 3917 using only waste beer yeast as nutrient source. *Bioresour. Technol.* 151 113–119. 10.1016/j.biortech.2013.10.052 24212131

[B42] Mohammadkazemi F. Azin M. Ashori A. (2015). Production of bacterial cellulose using different carbon sources and culture media. *Carbohydr. Polym.* 117 518–523. 10.1016/j.carbpol.2014.10.008 25498666

[B43] Moosavi-Nasab M. Yousefi M. (2011). Biotechnological production of cellulose by *Gluconacetobacter xylinus* from agricultural waste. *Iran. J. Biotechnol.* 9 94–101.

[B44] Nguyen V. T. Flanagan B. Mikkelsen D. Ramirez S. Rivas L. Gidley M. J. (2010). Spontaneous mutation results in lower cellulose production by a *Gluconacetobacter xylinus* strain from Kombucha. *Carbohydr. Polym.* 80 337–343. 10.1016/j.carbpol.2009.11.019

[B45] Nogi M. Yano H. (2008). Transparent nanocomposites based on cellulose produced by bacteria offer potential innovation in the electronics device industry. *Adv. Mater.* 20 1849–1852. 10.1002/adma.200702559

[B46] Park S. U. Lee B. K. Kim M. S. Park K. K. Sung W. J. Kim H. Y. (2014). The possibility of microbial cellulose for dressing and scaffold materials. *Int. Wound J.* 11 35–43. 10.1111/j.1742-481X.2012.01035.x 22762434 PMC7950980

[B47] Pascoal Neto C. Freire C. S. R. Fernandes S. C. M. Silvestre A. J. D. Gandini A. (2011). *Aqueous Coating Compositions for Use in Surface Treatment of Cellulosic Substrates.* Patent WO/2011/012934.

[B48] Petersen N. Gatenholm P. (2011). Bacterial cellulose-based materials and medical devices: current state and perspectives. *Appl. Microbiol. Biotechnol.* 91 1277–1286. 10.1007/s00253-011-3432-y 21744133

[B49] Pinheiro H. M. Touraud E. Thomas O. (2004). Aromatic amines from azo dye reduction: status review with emphasis on direct UV spectrophotometric detection in textile industry wastewater. *J. Dyes Pigm.* 61 121–139. 10.1016/j.dyepig.2003.10.009

[B50] Puppi D. Chiellini F. Piras A. M. Chiellini E. (2010). Polymeric materials for bone and cartilage repair. *Prog. Polym. Sci.* 35 403–440. 10.1016/j.progpolymsci.2010.01.006

[B51] Rangaswamy B. E. Vanitha K. P. Hungund B. S. (2015). Microbial cellulose production from bacteria isolated from rotten fruit. *Int. J. Polym. Sci.* 2015:280784. 10.1155/2015/280784 22391690

[B52] Rani M. U. Appaiah K. A. (2013). Production of bacterial cellulose by *Gluconacetobacter hansenii* UAC09 using coffee cherry husk. *J. Food Sci. Technol.* 50 755–762. 10.1007/s13197-011-0401-5 24425978 PMC3671040

[B53] Rethwisch D. G. Jr. William D. C. (2016). *Ciência e Engenharia de Materiais: Uma Introdução* 9th Edn. Rio de Janeiro: LTC.

[B54] Rivas B. Moldes A. B. Domínguez J. M. Parajó J. C. (2004). Development of culture media containing spent yeast cells of *Debaryomyces hansenii* and corn steep liquor for lactic acid production with *Lactobacillus rhamnosus*. *Int. J. Food Microbiol.* 97 93–98. 10.1016/j.ijfoodmicro.2004.05.006 15527923

[B55] Ross P. Mayer R. Benziman M. (1991). Cellulose biosynthesis and function in bacteria. *Microbiol. Rev.* 55 35–58. 2030672 10.1128/mr.55.1.35-58.1991 PMC372800

[B56] Sani A. Dahman Y. (2010). Improvements in the production of bacterial synthesized biocellulose nanofibres using different culture methods. *J. Chem. Technol. Biotechnol.* 85 151–164.

[B57] Santos D. K. F. Rufino R. D. Luna J. M. Santos V. A. Sarubbo L. A. (2016). Biosurfactants: multifunctional biomolecules of the 21st century. *Int. J. Mol. Sci.* 17 1–31. 10.3390/ijms17030401 26999123 PMC4813256

[B58] Santos S. M. Carbajo J. M. Quintana E. Ibarra D. Gomez N. Ladero M. (2015). Characterization of purified bacterial cellulose focused on its use on paper restoration. *Carbohydr. Polym.* 116 173–181. 10.1016/j.carbpol.2014.03.064 25458287

[B59] Shah J. Brown R. M. (2005). Towards electronic paper displays made from microbial cellulose. *Appl. Microbiol. Biotechnol.* 66 352–355. 10.1007/s00253-004-1756-6 15538556

[B60] Shah N. Ul-Islam M. Khattak W. A. Park J. K. (2013). Overview of bacterial cellulose composites: a multipurpose advanced material. *Carbohydr. Polym.* 98 1585–1598. 10.1016/j.carbpol.2013.08.018 24053844

[B61] Shao Y. Yashiro T. Okubo K. Fujii T. (2015). Effect of cellulose nano fiber (CNF) on fatigue performance of carbono fiber fabric composites. *J. Compos. A Appl. Sci. Manuf.* 76 244–254. 10.1016/j.compositesa.2015.05.033

[B62] Sheykhnazaria S. Tabarsaa T. Ashorib A. Shakeric A. Golalipourd M. (2011). Bacterial synthesized cellulose nanofibers; effects of growth times and culture mediums on the structural characteristics. *Carbohydr. Polym.* 86 1187–1191. 10.1016/j.carbpol.2011.06.011

[B63] Shi Z. Zhang Y. Phillips G. O. Yang G. (2014). Utilization of bacterial cellulose in food. *Food Hydrocoll.* 35 539–545. 10.1016/j.foodhyd.2013.07.012

[B64] Son C. Chung S. Lee J. Kim S. (2002). Isolation and cultivation characteristics of *Acetobacter xylinum* KJ-1 producing bacterial cellulose in shaking cultures. *J. Microbiol. Biotechnol.* 12 722–728.

[B65] Svensson A. Nicklasson E. Harrah T. Panilaitis B. Kaplan D. L. Brittberg M. (2005). Bacterial cellulose as a potential scaffold for tissue engineering of cartilage. *J. Biomater.* 26 419–431. 10.1016/j.biomaterials.2004.02.049 15275816

[B66] Tang W. Jia S. Jia Y. Yin H. (2014). Research on medical application of bacterial cellulose as nano-biomaterials. *Sheng Wu Yi Xue Gong Cheng Xue Za Zhi* 31 927–929. 25464815

[B67] Tanskul S. Amornthatree K. Jaturonlak N. (2013). A new cellulose-producing bacterium, *Rhodococcus* sp. MI 2: screening and optimization of culture conditions. *Carbohydr. Polym.* 92 421–428. 10.1016/j.carbpol.2012.09.017 23218315

[B68] Torres F. G. Commeaux S. Troncoso O. P. (2012). Biocompatibility of bacterial cellulose based biomaterials. *J. Funct. Biomater.* 3 864–878. 10.3390/jfb3040864 24955750 PMC4030925

[B69] Trovatti E. Serafim L. S. Freire C. S. R. Silvestre A. J. D. Pascoal Neto C. (2011). *Gluconacetobacter sacchari*: an efficient bacterial cellulose cell-factory. *Carbohydr. Polym.* 86 1417–1420. 10.1016/j.carbpol.2011.06.046

[B70] Tyagi N. Suresh S. (2016). Production of cellulose from sugarcane molasses using *Gluconacetobacter*: optimization and characterization. *J. Clean. Prod.* 112 71–80. 10.1016/j.jclepro.2015.07.054

[B71] Ul-Islam M. Khan T. Park J. K. (2012). Water holding and release properties of bacterial cellulose obtained by in situ and ex situ modification. *Carbohydr. Polym.* 88 596–603. 10.1016/j.carbpol.2012.01.006

[B72] Vazquez A. Foresti M. L. Cerrutti P. Galvagno M. (2013). Bacterial cellulose from simple and low cost production media by *Gluconacetobacter xylinus*. *J. Polym. Environ.* 21 545–554. 10.1007/s10924-012-0541-3

[B73] Wu D. Li X. Shen C. Lu J. Chen J. Xie G. (2014). Decreased ethyl carbamate generation during Chinese rice wine fermentation by disruption of CAR1 in an industrial yeast strain. *Int. J. Food Microbiol.* 180 19–23. 10.1016/j.ijfoodmicro.2014.04.007 24769164

